# Cytosolic 5’-Nucleotidase II Interacts with the Leucin Rich Repeat of NLR Family Member Ipaf

**DOI:** 10.1371/journal.pone.0121525

**Published:** 2015-03-26

**Authors:** Federico Cividini, Maria Grazia Tozzi, Alvaro Galli, Rossana Pesi, Marcella Camici, Charles Dumontet, Lars Petter Jordheim, Simone Allegrini

**Affiliations:** 1 University of Pisa, Department of Biology, Biochemistry Unit, Pisa, Italy; 2 National Research Counsil (CNR), Institute of Clinical Physiology, Pisa, Italy; 3 University of Sassari, Department of Chemistry and Pharmacology, Sassari, Italy; 4 Université de Lyon, Lyon, France; 5 Université de Lyon 1, Lyon, France; 6 INSERM U1052, Centre de Recherche en Cancérologie de Lyon, Lyon, France; 7 CNRS UMR 5286, Centre de Recherche en Cancérologie de Lyon, Lyon, France; International Centre for Genetic Engineering and Biotechnology, ITALY

## Abstract

IMP/GMP preferring cytosolic 5'-nucleotidase II (cN-II) is a bifunctional enzyme whose activities and expression play crucial roles in nucleotide pool maintenance, nucleotide-dependent pathways and programmed cell death. Alignment of primary amino acid sequences of cN-II from human and other organisms show a strong conservation throughout the entire vertebrata taxon suggesting a fundamental role in eukaryotic cells. With the aim to investigate the potential role of this homology in protein-protein interactions, a two hybrid system screening of cN-II interactors was performed in *S*. *cerevisiae*. Among the X positive hits, the Leucin Rich Repeat (LRR) domain of Ipaf was found to interact with cN-II. Recombinant Ipaf isoform B (lacking the Nucleotide Binding Domain) was used in an *in vitro* affinity chromatography assay confirming the interaction obtained in the screening. Moreover, co-immunoprecipitation with proteins from wild type Human Embryonic Kidney 293 T cells demonstrated that endogenous cN-II co-immunoprecipitated both with wild type Ipaf and its LRR domain after transfection with corresponding expression vectors, but not with Ipaf lacking the LRR domain. These results suggest that the interaction takes place through the LRR domain of Ipaf. In addition, a proximity ligation assay was performed in A549 lung carcinoma cells and in MDA-MB-231 breast cancer cells and showed a positive cytosolic signal, confirming that this interaction occurs in human cells. This is the first report of a protein-protein interaction involving cN-II, suggesting either novel functions or an additional level of regulation of this complex enzyme.

## Introduction

Cytosolic 5’-nucleotidase II (cN-II) is an IMP/GMP preferring 5’-nucleotidase whose activity, structure and expression has drawn the attention of several research groups from various fields such as biochemistry, crystallography, molecular biology, oncology, pharmacology and genetics. cN-II is a bifunctional enzyme [1, 2], operating both as a phosphatase and a phosphotransferase, and these activities might contribute to the maintenance of the qualitative and quantitative balance of intracellular purine compounds [3]. The enzyme has been purified from different sources showing an ubiquitous distribution, a very high primary sequence conservation and complex allosteric regulation [4–6]. Among the enzymes belonging to the purine and pyrimidine metabolic pathways only hypoxanthine-guanine phosphoribosyl transferase (HGPRT) exhibits a comparable primary sequence conservation [7] which probably reflects additional roles out in cell physiology. Indeed, HGPRT gene serves not only to drive classical purine salvage pathway, but also to regulate multiple key functions in neuronal development [8–11].

Fluctuations of cN-II expression and activity have been associated with highly proliferating cells and neurological disorders [12, 13], and mutations in *NT5C2* (the gene encoding cN-II) have been reported to drive pharmacological resistance in hematological tumors [14, 15]. Moreover, clinical and preclinical observations have led to the hypothesis that 5′-nucleotidase cN-II could constitute a therapeutic target in oncology [16]. In 2008, our group demonstrated that cN-II is fundamental for human glioblastoma ADF cell line survival which underwent apoptosis as soon as cN-II expression and activity decreased below 40% of the baseline levels [17]. This finding, which is at least not obviously directly linked to the enzymatic activity of cN-II, suggests that cN-II is implicated in cellular processes outside nucleotide metabolism, and such activities could be dependent on either direct or indirect interaction with other proteins.

**Fig 1:**
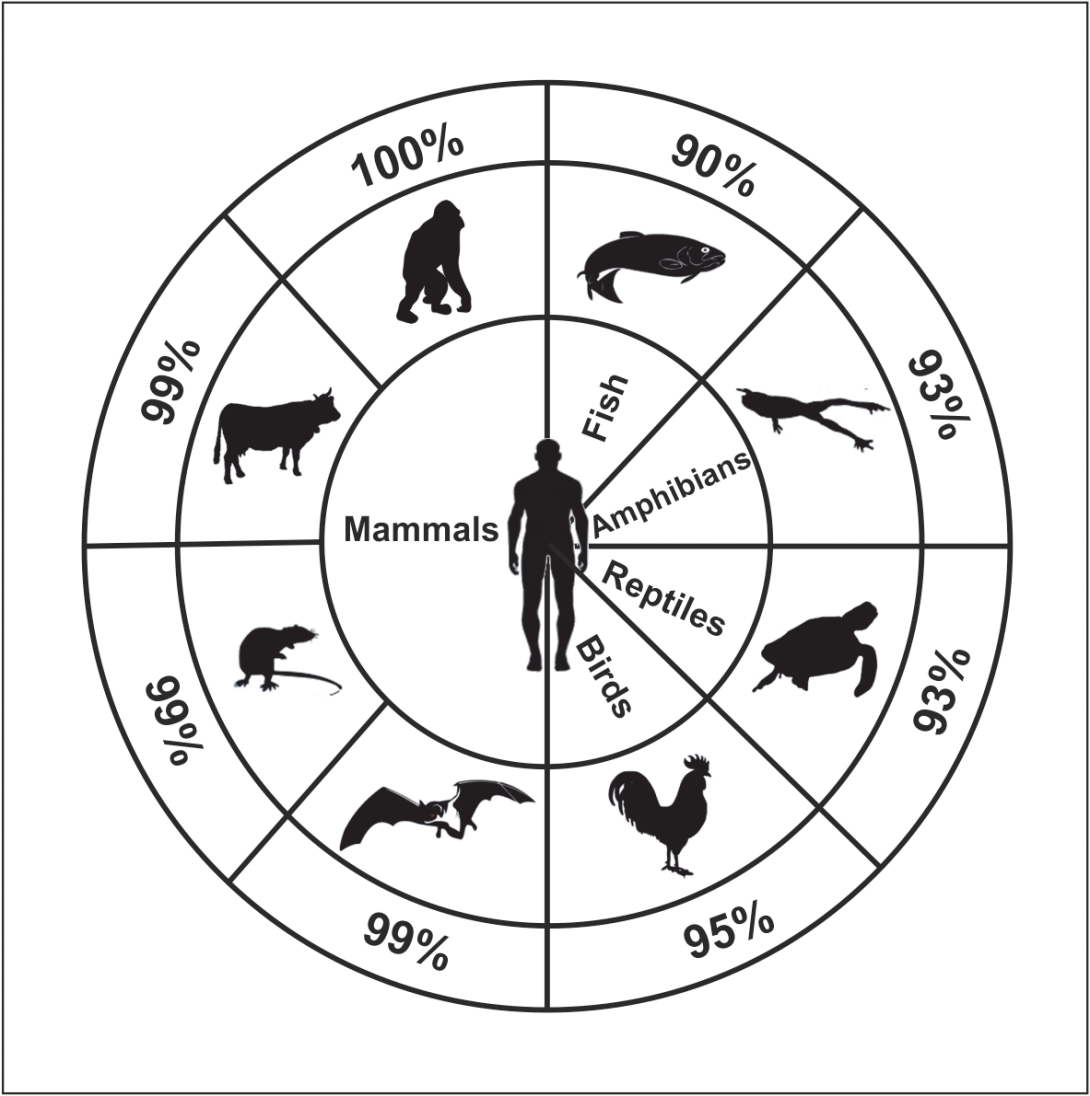
Percentage of protein homology obtained from UniProt Web Site (http://www.uniprot.org/) by blasting human cytosolic 5’-nucleotidase II primary sequence (P49902) with others belong to different vertebrates: *Pongo abelii* (Q5RA22), *Bos Taurus* (B1H0W4), *Mus musculus* (E9Q9M1), *Myotis brandtii* (S7PW19), *Gallus gallus* (F1NCR3), *Chelonia mydas* (M7BZT7), *Xenopus laevis* (Q6DKB0) and *Dario rerio* (F1QAK5).

The widespread distribution and the extreme conservation among vertebrates of the protein sequence (Fig. 1) may suggest a fundamental role played by this enzyme in vital cell functions. Based on this sequence conservation and new potential roles for cN-II in the cell, we performed a two hybrid system-based screening to identify cN-II interactors. We present evidence demonstrating that cN-II interacts with the leucin rich domain (LRR) of the Nod-like receptor Ipaf (Ice protease-activating factor, also known as NLRC4, CLAN or CARD12) both *in vitro* and in human cells.

We believe that further studies of the interaction between cN-II and Ipaf could contribute to a better understanding of the innate immune response, as well as to new regulatory roles of cN-II in cell biology.

## Materials and Methods

### Plasmid and cloning

All cloning was performed according to standard procedures [18]. Plasmid carrying bovine cN-II (pET-28cDNA-cN-II) was previously described [19]. The plasmid used in the two hybrid screening was constructed by cloning the PCR-amplified cDNA of cN-II into the pBD-Gal-Cam vector (Agilent, Santa Clara, USA) to form pBD-Gal-cNII [20]. The human cDNA library (Mate & Plate^TM^ Library-Universal Human (Normalized), Clontech) was cloned into the pGADT7-RecAB vector (Clontech, Jesi, Italy). Plasmids pReceiver.B01, carrying the complete cDNA for the NOD-like receptor Ipaf (pRec.B01-Ipaf), pEZ-M15-YFP/Ipaf and pEZ-M02-Ipaf were purchased from GeneCopoeia (Rockville, USA) and verified by restriction fragments analysis and sequencing (BIO-FAB research, Rome, Italy). Plasmid for Ipaf isoform B was obtained by deletion of the cDNA encoding the NBD domain in pRec.B01-Ipaf. This was performed by amplification of the LRR and CARD domains using two semi-complementary primers: T7 Fwd together with 5'-AGTCAGACCACTTTGTCCATTCAAGTCC-3', and B01 Rev together with 5'-GGACAAAGTGGTCTGACTGAC AGCTT-3', respectively. Fragments were hybridized and amplified with T7 fwd and B01 rev. PCR products and pRec.B01-Ipaf were digested with NotI and XbaI (New England Biolabs_,_ Ipswich, USA), separated in 0.8% agarose gel, purified with “Wizard SV Gel” and “PCR Clean-Up System” (Promega, Milan, Italy) and subsequently ligated (“Rapid DNA ligation kit” Roche, Milan, Italy) to form pReceiver.B01-IpafB. All procedures were conducted following the manufacturer instructions. pRec.B01-IpafB was controlled by PCR analysis with T7 prom forward primer and B01 reverse primer, restriction fragments analysis and sequencing (Bio-Fab). Plasmids pEGFP-C1-Ac-Ipaf and pcDNA3.1-myc-LRR were kindly provided by Pr. G. Swarup [20]. In our experiments the cDNA encoding the LRR domain was expressed after subcloning the cDNA in the vector pEGFP-C1 (Clontech) in frame with GFP using EcoRI and XbaI (New England Biolabs, Ipswich, USA) following the manufacturer instructions. For decreased cN-II expression, a specific sequence for cN-II was determined at position 349, and the following oligomers were purchased from Dharmacon (target sequence underlined): Top 5’-GATCCCCAACCTCTTGGTCTGTGCACATTTCAAGAGAATGTGCACA GACCAAGAGGTTTTTTTGGAAA-3’, bottom 5’-TCGATTTCCAAAAAAACCTCTTGGTCTGTGCACATTCTCTTGA AATGTGCACAGACCAAGAGGTTGGG-3’, hybridized and cloned into pSUPERIOR.neo (OligoEngine, Seattle, USA) with BglII and XhoI to obtain the pScN-II vector. A non-targeting control plasmid (pScont) was prepared in the same way with the following oligomers: Top 5’-GATCCCCCACTACCGTTGTTAAGGTGTTC AAGAGACACCTTAACAACGGTAGTGTTTTTA-3’, bottom 5’-AGCTTAAAAACACTACCGTTGTTAAGGTGTCTC TTGAACACCTTAACAACGGTAGTGGGG-3’.

### Two-hybrid screening

The pGADT7-RecAB vector carrying the human cDNA library was transformed in the *Saccharomyces cerevisiae* strain Y187 (*MAT*α, *ura3-52*, *his3-200*, *ade2-101*, *trp1-901*, *leu2-3*, *112*, *gal4*Δ, *met-*, *gal80*Δ, *MEL1*, *URA3*: *GAL1UAS-GAL1TATA*-*lacZ)* by standard procedure using lithium acetate and ssDNA as carrier [21]. Yeast media and culturing was performed as previously reported [20]. The *S*. *cerevisiae* strain AH109 (*MATa*, *trp1-901*, *leu2-3*, *112*, *ura3-52*, *his3-200*, *gal4Δ*, *gal80Δ*, *LYS2*: *GAL1UAS-GAL1TATA-HIS3*, *GAL2UAS-GAL2TATA-ADE2*, *URA3*: *MEL1UAS-MEL1TATA-lacZ*) was transformed with pBD-GAL-cN-II. Screening was carried out by mating the two yeast strains in complete liquid medium for 17 hours at 30°C under shaking, plating the culture in the medium lacking tryptophan and leucine to select for diploids (for details see the manual from Clontech). In parallel, the culture containing about 10^9^ cells was plated onto medium lacking tryptophan, leucine, adenine and histidine to score for positive clones. Then, positive clones were streaked out onto medium lacking tryptophan, leucine, adenine and histidine to confirm the positiveness. Clones were cultivated in medium lacking leucine for 48 hours at 30°C and plasmids were extracted using the yeast plasmid miniprep kit (Zymo Research Orange, Irvine, USA) and transformed in competent *E*. *coli* for amplification. Plasmid DNA was extracted from *E*. *coli* by standard method and further analyzed. The cDNA from plasmids extracted from all positive clones was sequenced.

### Expression of recombinant cN-II and Ipaf B

*E*. *coli* strains BL21 Rosetta (DE3) and BL21 (DE3) were used for Ipaf B and cN-II protein expression respectively, with transformation as described before [19]. The expression of the recombinant protein was performed in LB medium containing 1 mM isopropyl β-D-thiogalactoside (IPTG) and 1 mg/ml of ampicillin at 20°C overnight on a shaking plate.

### Affinity chromatography

After overnight growth, bacteria were harvested and the pellet was suspended in lysis buffer (50 mM Tris-HCl pH 7.4, 300 mM NaCl, Protease Inhibitors Cocktail-ICN Biomed. Inc., Milan, Italy). Lysis was achieved by freeze-thawing the cells in the presence of 2 mg/ml lysozyme. Lysates were then centrifuged at 35,000 g for 90 min. Supernatant from *E*. *coli* BL21 (DE3) containing cN-II was loaded onto the “Perfect Pro Ni-NTA Agarose” beads (5’Prime Inc, Milan, Italy) equilibrated in the lysis buffer. The beads were then washed with 10 column volumes of lysis buffer, 100 mM NaCl, 30 mM imidazole in 20 mM Tris-HCl, pH 8.0. Successively, supernatant from *E*. *coli* BL21 Rosetta (DE3) expressing Ipaf B was loaded onto the same Ni-NTA beads. Absorbance at 280 nm was monitored and when flow-through proteins stopped eluting the beads were washed with 10 volumes of 10 mM Tris-HCl pH 7.4 and finally recombinant proteins were eluted from the bead with 250 mM imidazole in 50 mM Tris-HCl, pH 7.4. As control, the same experiment was performed without loading of supernatant from *E*. *coli* BL21 (DE3) with cN-II.

### Cell culture and cell transfection

All cell lines were from ATCC and cultured in Dulbecco's modified Eagle's medium (DMEM) supplemented with 10% Fetal Bovine Serum, 100 U/ml penicillin and 100 μg/ml streptomycin (LONZA, Basel, Switzerland) at 37°C in a humidified 5% CO_2_ / 95% air atmosphere. Transfections were performed with plasmids previously expanded and purified from *E*. *coli* DH5α using the The PureLink HiPure Plasmid Filter Maxiprep Kit (Invitrogen). A549 and MDA-MB-231 cells were stably transfected with pScN-II and pScont using lipofectin. Briefly, cells (200,000 cells per T25 flask) were incubated for 5 hours with 5 μg of plasmid in presence of 12.5 μg of lipofectin (Invitrogen). After 72 h of growth in complete medium stably transfected cells were selected for three to four weeks with appropriate concentrations of neomycin (0.8 mg/ml). For transient transfection of HEK 293 T and MCF7 cells 70% confluent T75 flask were incubated for 5 hours with 15 μg of plasmids in presence of 37.5 μg of lipofectin (Invitrogen, Monza, Italy) before complete medium was added.

### Co-immunoprecipitation

Transiently transfected HEK 293 T cells were harvested and pellets were suspended in lysis buffer (20 mM Tris-HCl pH 6.8, 1 mM MgCl_2_, 2 mM EGTA, 0.5% NP40, proteases inhibitors cocktail). Cells were disrupted by repeating aspiration trough a 21 gauge needle and incubated for 1 h at 4°C. Cellular debris were pelleted by centrifugation at 12000 g for 15 min at 4°C and 500 μg of proteins were incubated three times for 30 min with 20 μl of A/G plus-Agarose (Santa Cruz Biotechnology, Inc., Heidelberg, Germany) previously equilibrated with lysis buffer without proteases inhibitors. After each incubation, samples were centrifuged at 2500 g for 5 min and beads were discarded. The remaining protein sample was incubated for 2 h with 2 μg of primary antibody (Anti cN-II, clone 3C1, 1/500, Abnova, Jhongli City, Taiwan) and subsequently incubated at 4°C on a rocker platform rotating device overnight after adding 20 μl of A/G plus-Agarose. Immunoprecipitates were collected by centrifugation at 2500 g for 5 min and washed three times with 1 ml of lysis buffer without proteases inhibitors. Finally, pellet was suspended in 50 μl of electrophoresis sample buffer and incubated for 5 min at 95°C, centrifuged at 2500 g for 5 min. A second elution with 15 μl was performed and the total amount of 65 μl of eluted proteins was separated by SDS-PAGE and analyzed with western and immunoblotting.

### Western and immunoblot analysis

Proteins were extracted from cell pellets as described elsewhere [22]. Protein content was determined by the Bradford assay using BSA (Sigma, Milan, Italy) as standard, and proteins were separated by SDS-PAGE using 10% acrylamide and transferred onto nitrocellulose membrane using iBlot system (Life Technologies, Monza, Italy). Membranes were incubated with specific antibodies for cN-II (clone 3C1, 1/500, Abnova), GFP (ab290, 1/2000, Abcam, Cambridge, UK), Ipaf (Anti CARD12 polyclonal ab in Rabbit, 1/500, Novus Biological, Cambridge, UK), and β-actin (clone AC-15, 1/5000, Sigma). Secondary anti-murine (IRdye 800CW, 1/5000, LI-COR Biosciences, Lincoln, USA) or anti-rabbit antibody (IRdye 680, 1/5000, LI-COR Biosciences) were used and protein expression was visualized using the Odyssey infrared system (LI-COR Biosciences).

### Proximity Ligation Assay

Cells (10,000 per well) were seeded on Nunc Lab-Tek Chamber Slide System (ThermoScientific, Walham, USA). Transient transfection of MCF7 cells was conducted for 48 h directly on the same support. After 48 h of transfection (for MCF7 cells) or the day after seeding (for A549 and MDA-MB-231 cells), medium was removed from the chambers and cells were washed twice with PBS 1X, fixed with 4% PFA and permeabilized with 0.1% Triton in PBS 1X. Unspecific proteins were blocked with PBS 1X, FBS 1% and BSA 0.1%. Cells were then incubated with primary antibodies against cN-II and Ipaf at 4°C overnight. The day after, cells were incubated with the appropriate DNA-linked secondary antibody furnished by the Duolink kit (Olink Bioscience, St. Louis, USA) and PCR *in situ* amplification was performed using the PLA technology according to the manufacturer’s instructions. The PLA signal was detected with fluorescence microscopy and images were acquired at the Centre d’Imagerie Quantitative Lyon Est (University Claude Bernard Lyon 1, Lyon, France) using a Confocal Laser Scanning Microscope (Leica SP2 CLSM, Heidelberg, Germany) equipped with an x63 objective lens (NA 1.32). The acquisition process contained three steps. First, images of GFP were acquired with an excitation wavelength of 488 nm and collected from 580 nm to 658 nm. Secondly, images of Cy3 staining were acquired with an excitation wavelength of 641 nm and collected from 678 nm to 703 nm. Finally, images of DAPI staining were acquired with an excitation wavelength of 405 nm and collected from 429 nm to 498 nm. As a technical control the same protocol was applied to cells exposed either only to one primary antibody or none of them.

## Results

### Identification of Ipaf as cN-II possible interactor

The high percentages of homology between cN-II from human and from other vertebrates (Fig. 1) suggested the possibility that its whole structure could be important for its overall activity and potentially through interacting with other proteins. Thus, to identify cN-II-interacting proteins, a Gal4-based yeast two-hybrid screening of a human placental cDNA library was performed in *S*. *cerevisiae*, using the complete cN-II (1–561 aa) as bait. The screening was performed twice and gave 39 and 29 positive clones respectively. When eliminating clones that could not be sequenced, that contained mutations in the activation domain or that were not positive in the growth on medium lacking leucine, only 18 clones remained from the first screen and 29 from the second (see S1 Table). The majority of these encoded only few aminoacids or short peptides with no significant similarity found in blast search. One protein was identified as a cN-II interactor in both experiments (see S1 Table) with the identification of 172 and 263 aminoacids. The last clone, identified in the second experiment, encoded 97 aminoacids of the LRR domain of Ipaf. Based on recent results on the role of Ipaf in induction of apoptosis and the observation that cN-II inhibition can lead to cell death, we chose to continue here with the study on the interaction between Ipaf and cN-II.

### In vitro interaction between recombinant cN-II and IpafB

**Fig 2:**
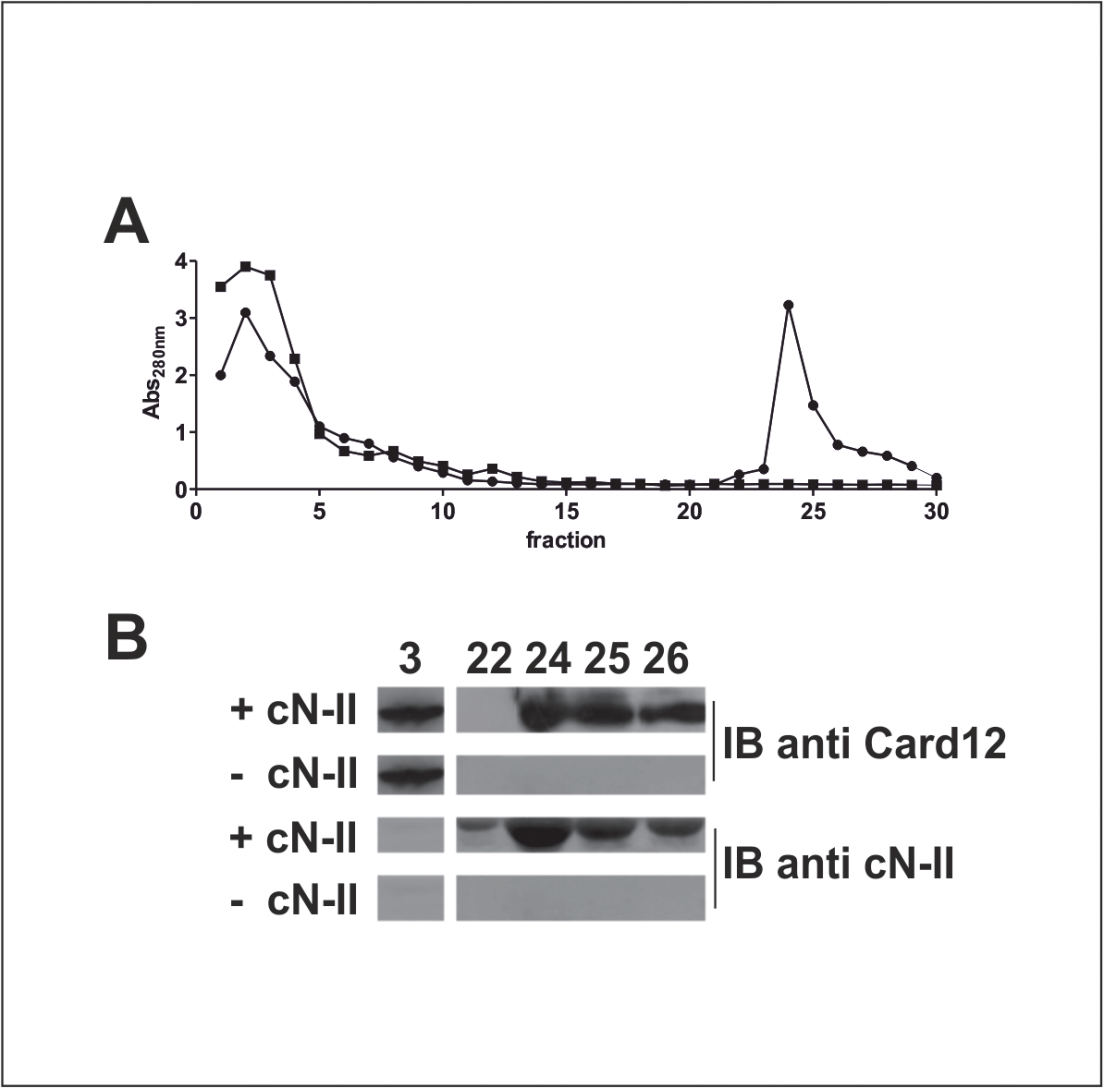
*In vitro* interaction between cN-II and Ipaf B. After loading Ni-talon beads with *E*. *coli* “BL21(DE3)” crude extract without (■) or with cN-II (●), beads were washed and *E*. *coli* “BL21 Rosetta(DE3)” extracts containing Ipaf B were loaded. Elution buffer containing 250 mM imidazole was used from fraction 21 to detach His-tagged cN-II and elute proteins. A) proteins elution profile; B) immunoblotting with CARD12 and cN-II antibodies on elution fractions 3, 22, 24, 25 and 26 from chromatography.

In order to confirm this interaction between cN-II and Ipaf, we expressed both recombinant proteins and verified their *in vitro* interaction using an affinity chromatography. Due to difficulties of expressing Ipaf in several *E*. *coli* strains we deleted the nucleotide binding domain and performed experiments with the 40 kDa isoform B of Ipaf, whereas cN-II was attached to a His-tag in order to interact with the beads used for chromatography. Evidence of recombinant expression of Ipaf-B in *E*. *coli* strain upon IPTG induction is reported in S1 Fig, whereas cN-II was expressed as reported elsewhere [19]. The elution profile of proteins showed that, after the elimination of unbound proteins within the first 10 fractions, proteins are detected in fractions 24–26 (Fig. 2A). Western and immunoblot analysis of fractions 24–26 confirmed the presence of both cN-II and Ipaf B indicating that Ipaf B was retained by cN-II and co-eluted with this protein (Fig. 2B). On the contrary, crude extracts containing Ipaf B did not bind the Ni-NTA beads that were not preloaded with extracts containing cN-II as shown both by the elution profile and the immunoblot analysis. These data suggest that Ipaf-B were retained in the chromatography through interaction with cN-II.

### cN-II interacts with Ipaf in human cell extracts

**Fig 3:**
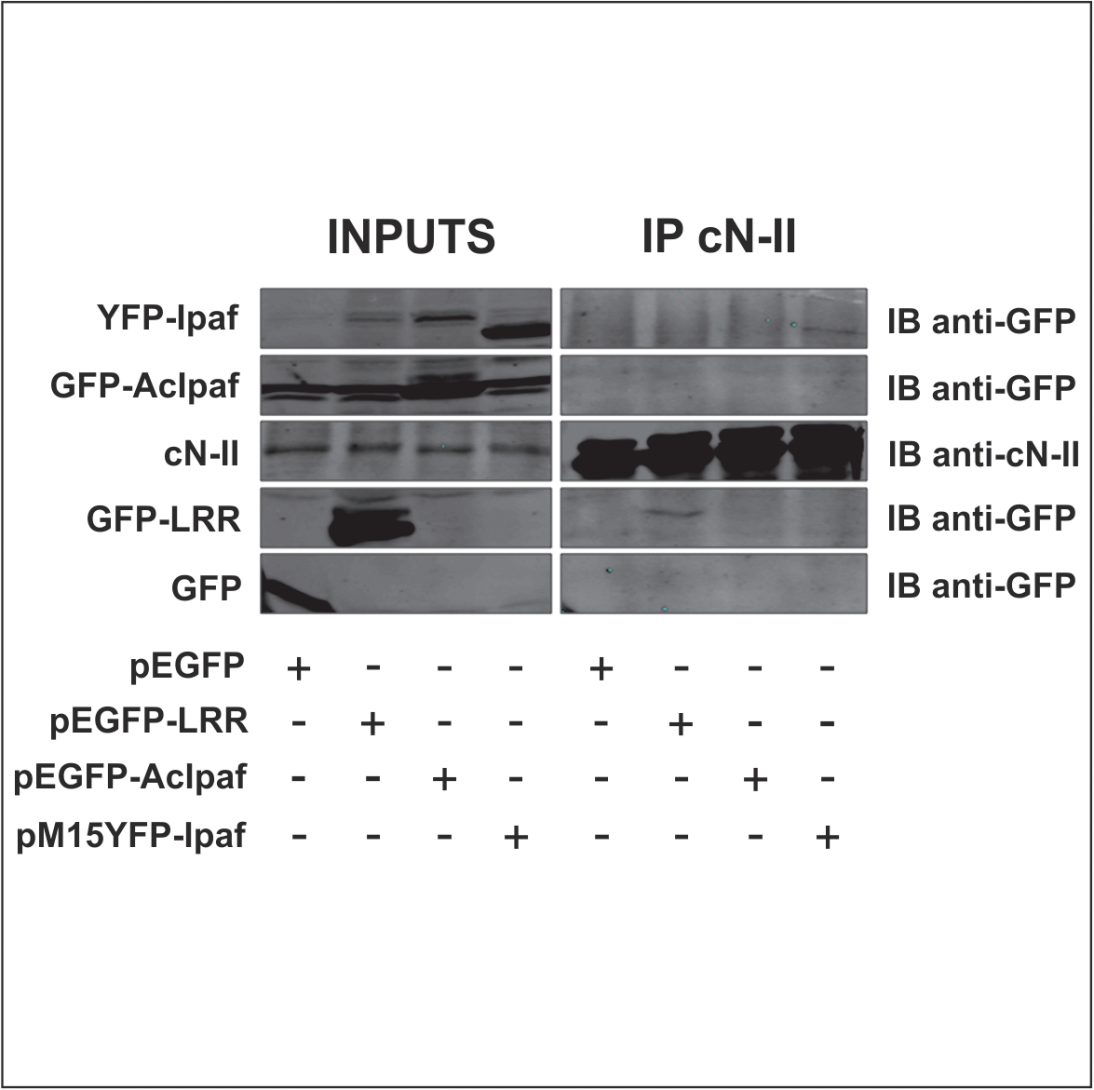
Co-immunoprecipitation cN-II and variants of Ipaf from HEK 293 T cells. Inputs show expression of indicated proteins in cell extracts from cells transfected with plasmids indicated under the figure. IP cN-II represents proteins that were present after immunoprecipitation with cN-II antibody on cell extracts from cells transfected with indicated plasmids.

We further investigated whether the interaction between cN-II and Ipaf could be detected in human cells. We screened for the protein expression of Ipaf and cN-II in several cell lines (see S2 Fig) using THP-1 and MCF7 as positive and negative controls respectively for Ipaf expression [23, 24, 25]. We performed thereafter a co-immunoprecipitation assay in the Human Embryonic Kidney 293 T cell line as these are easily transfected. We transfected cells with plasmids encoding different forms of Ipaf fused to the green fluorescence protein (GFP) or to the yellow fluorescence protein (YFP) with the aim of either increase the amount of the whole protein within cytoplasm or mapping the region through which the interaction with cN-II takes place. GFP-fusion was used to tag transgene proteins using anti-GFP antibodies. Forty-eight hours after transfection, transgene expression was visually verified with fluorescent microscopy and cN-II was immunoprecipitated from the crude extract of cells. Exogenous forms of Ipaf were detected by immunoblotting (Fig. 3) using anti-GFP antibody. Results clearly show that endogenous cN-II is able to precipitate exogenous Ipaf (transfection with pM15YFP-Ipaf) and the LRR domain (pEGFP-LRR plasmid) fused to GFP or YFP, whereas both GFP alone (pEGFP plasmid) and AcIpaf (pEGFP-AcIpaf plasmid) lacking the LRR domain did not precipitate with cN-II. These results strongly suggest that the interaction between cN-II and Ipaf requires the LRR domain of Ipaf.

### cN-II interacts with Ipaf in human cells

**Fig 4:**
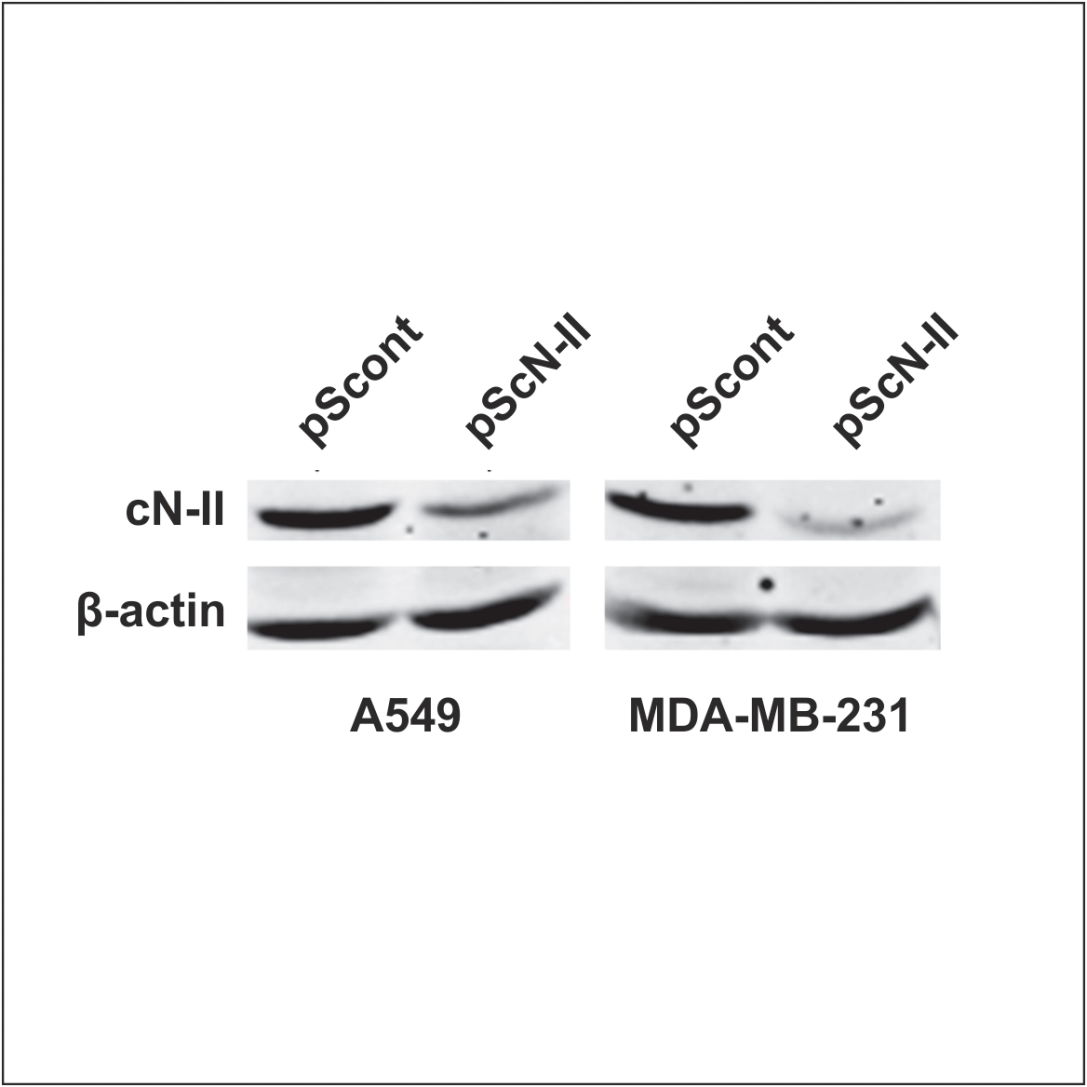
cN-II expression in A549 and MDA-MB-231 cells stably transfected with pScont or pScN-II. The immunoblot was performed on cell extracts from transfected cells and show the decreased cN-II expression in pScN-II transfected cells.

**Fig 5:**
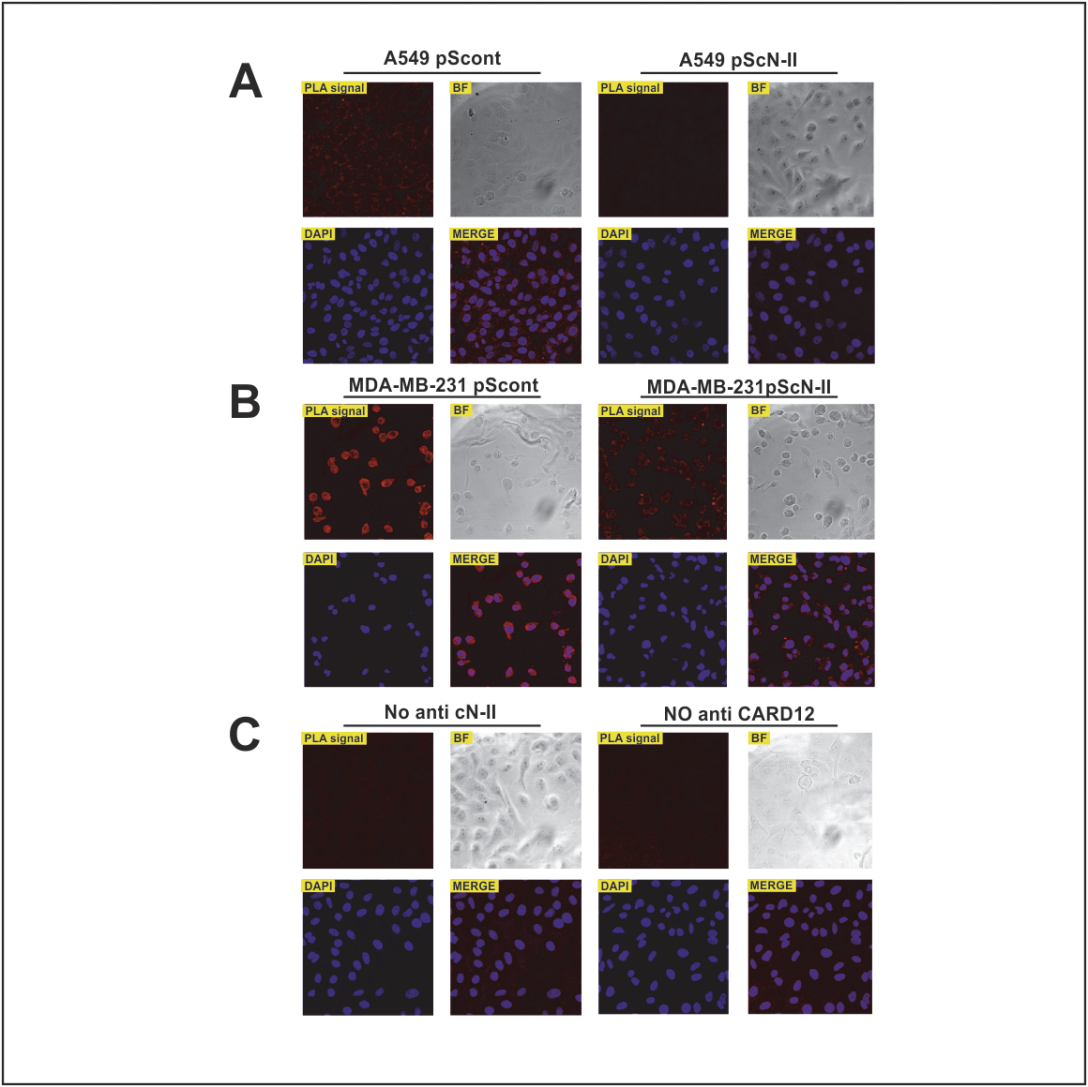
Proximity ligation assay for cN-II-Ipaf interaction in A549 (A) and MDA-MB-231 (B) cells. Stably transfected cells with pScont (control plasmid) or pScN-II (for cN-II downregulation) were used. Control assay was performed in A549 pScont using either only one anti-Ipaf or only anti-cN-II antibody (C). Red dots in PLA signal correspond to the detection of cN-II interacting with Ipaf; DAPI corresponds to the nuclear staining of the cells; BF is the image of the cells in bright field; Merge corresponds to the merging of PLA and DAPI images.

**Fig 6:**
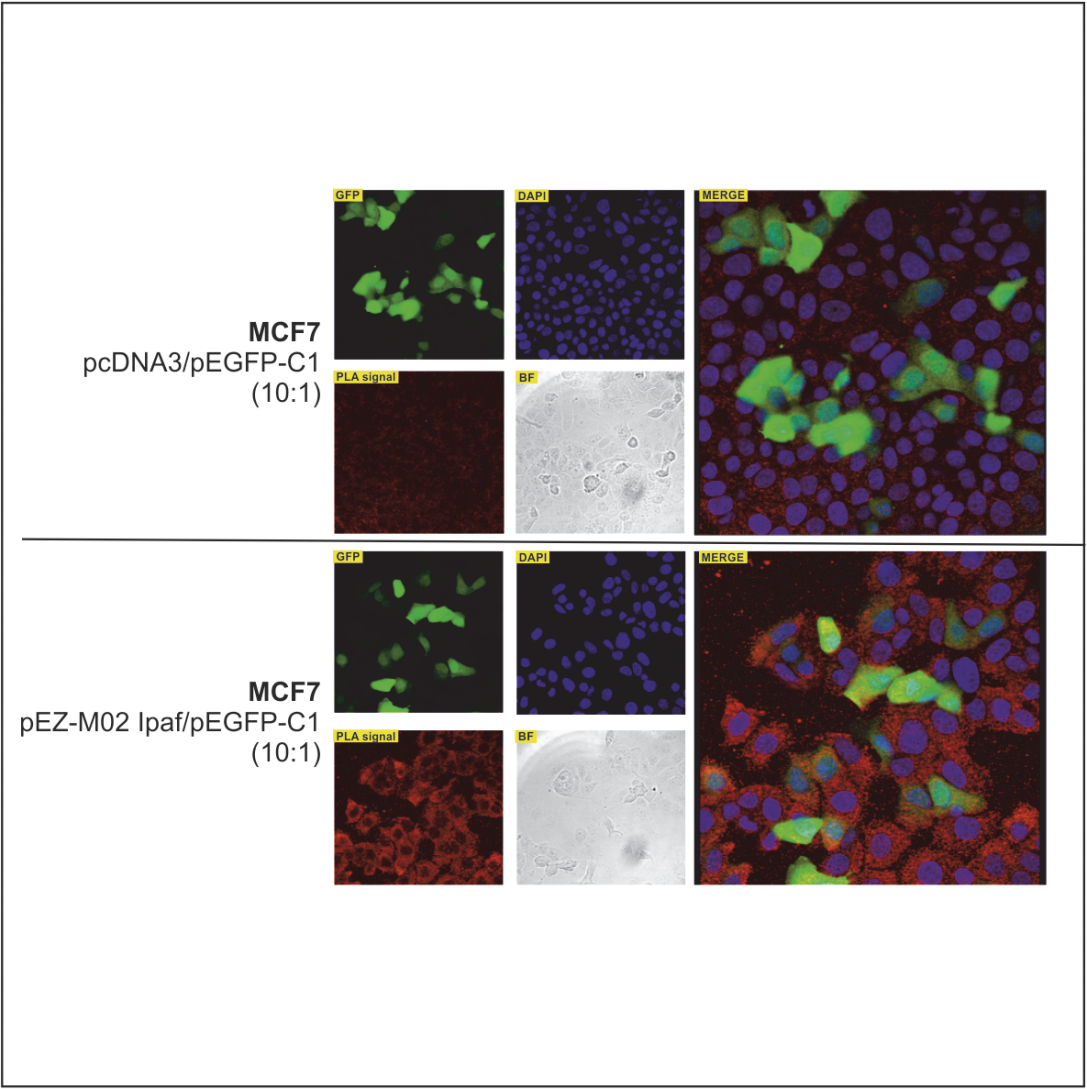
Proximity ligation assay for cN-II-Ipaf interaction in MCF7 cells. Cells were transiently co-transfected with the empty vector pcDNA3 and pEGFP-C1 (ratio 10:1, upper panel) or with pEZ-M02 Ipaf and pEGFP-C1 (ratio 10:1, lower panel) and subsequently analyzed for protein-protein interaction by PLA in GFP-positive cells. Red dots in PLA signal correspond to the detection of cN-II interacting with Ipaf; DAPI corresponds to the nuclear staining of the cells; BF is the image of the cells in bright field; GFP shows GFP-positive cells; Merge corresponds to the merging of PLA, DAPI and GFP images.

To confirm the interaction between the two proteins in intact cells, we used a PLA in human lung carcinoma cells (A549) and in human breast cancer cells (MDA-MB-231) since both interactors are well expressed in these cell models (see S2 Fig). With the aim to ascertain the specificity of this assay, we down-regulated cN-II expression by stable transfection with a shRNA-coding plasmid (Fig. 4) and verified that the PLA signal decreased upon silencing (Fig. 5). Results clearly demonstrate the specificity of this interaction since the signal generated is dependent on cN-II expression level in both cell lines (Fig. 5A and B). As technical control we performed the PLA assay in A549 stably transfected with pScont in the absence of either anti cN-II or anti Card12 antibodies, that revealed no PLA signal (Fig. 5C). We performed a similar experiment in breast cancer MCF7 cells which have very low endogenous expression of Ipaf (see S2 Fig). These cells were transiently co-transfected with plasmids encoding either whole Ipaf or GFP (ratio 10:1). Co-transfection with pEGFP was performed in order to continue analysis only on GFP-positive cells that would statistically also be transfected with Ipaf, whereas GFP-negative cells could be positive or negative for Ipaf. As a control, cells were co-transfected with the empty pcDNA3 vector together with pEGFP in the same ratio. Results from this experiment clearly show a stronger signal upon co-transfection with vectors carrying GFP and whole Ipaf as compared to GFP and pcDNA3 co-transfection (Fig. 6). In both experiments, PLA signal was detected in the cytoplasm and confirmed that interaction between cN-II and Ipaf also occurs in human cells.

## Discussion

The strong conservation of the primary cN-II sequence throughout the *vertebrata taxon* together with the growing body of data of multiple roles of cN-II in cell physiology, prompted us to investigate the possibility that cN-II could play a regulatory role dependent on protein-protein interactions in addition to its well-known catalytic activity. During the last decades, the catalytic function of cN-II has been extensively studied [26–29] and its expression has been modulated with the intent to characterize its impact on nucleotide pools inside cells [17, 30, 31]. Efforts have been made to investigate on the modality through which cN-II expression and activity causes resistance to different antimetabolites commonly used in anticancer therapies [32–37]. In some cases, increased cell proliferation or activation of an apoptotic pathway were observed upon over expression or silencing of cN-II [17, 30, 31], which contribute to consider cN-II as a possible regulative protein of major processes in the cell physiology. We therefore hypothesized that cN-II could influence on cell cycle through interaction with its checkpoints. Based upon information detailed in the introduction, such regulatory role could be dependent on protein-protein interactions.

We used a two hybrid system screening with the aim to detect possible cN-II interactors. The two independent screens performed identified several positive clones, but the majority turned out to be without interest. A clone containing the sequence corresponding to 97 aminoacids of the LRR domain of Ipaf was identified in the second run.

Ipaf belongs to the family of NBS-LRR (nucleotide-binding site and leucine-rich repeat) proteins and has been shown to take part in several intracellular signaling pathways involved in inflammatory responses. Four isoforms arising from alternate splicing of Ipaf mRNA have been identified in humans. The longest transcript termed Ipaf-A is 3.370 kb with an ORF encoding 1024 amino acids. Ipaf-B, C and D have exons corresponding to the nucleotide binding domain (NBD) spliced selectively to other exons forming shorter transcripts [38] which, in our knowledge, have not been shown to have any specific biological activity. The scientific literature on Ipaf mainly reports on the isoform A (named in this paper generically Ipaf) as an important character in innate immune responses to Gram-negative bacteria infection [39–41]. In many studies Ipaf has been reported to activate caspase-1 in macrophages infected with *Legionella pneumophila*, *Salmonella typhimurium*, *Shigella flexneri* or *Pseudomonas aeruginosa* leading to macrophage cell death and the release of proinflammatory cytokines [42]. In addition, Kumar *et al*. demonstrated an alternative pathway through which Ipaf leads to cell death by interaction with the component of the 26S proteasome Sug1 through caspase-8 recruitment and activation without any bacterial infection [23]. In the same work the authors demonstrate that despite several results suggesting the involvement of Ipaf in rapid release of proinflammatory cytokines in response to various microbial *stimuli*, other roles of this protein remain possible. In view of the roles of this protein in cell biology and in particular in cell survival and induction of cell death, we found it to be an interesting potential candidate to explain some of the more recently described roles of cN-II.

The interaction between cN-II and Ipaf was confirmed *in vitro* through co-purification of cN-II and a shortened form of IPAF. Furthermore co-immunoprecipitation experiments in HEK-293T demonstrated that Ipaf interacts with cN-II in human cell extracts through its LRR domain adding relevant information as to the interaction between proteins expressed and synthesized by human cells. The LRR domain is universally defined as the sensing domain through which Ipaf recognizes specific pathogen associated molecular patterns [43–45]. Finally PLA provided evidence that the interaction is specific and present in live human cells. Thus, the interaction between the two proteins has been clearly demonstrated by several approaches. This is to our knowledge the first demonstration of direct interaction between a key enzyme of the nucleotide metabolism and an intracellular pattern recognition receptor of the innate immune system.

**Fig 7:**
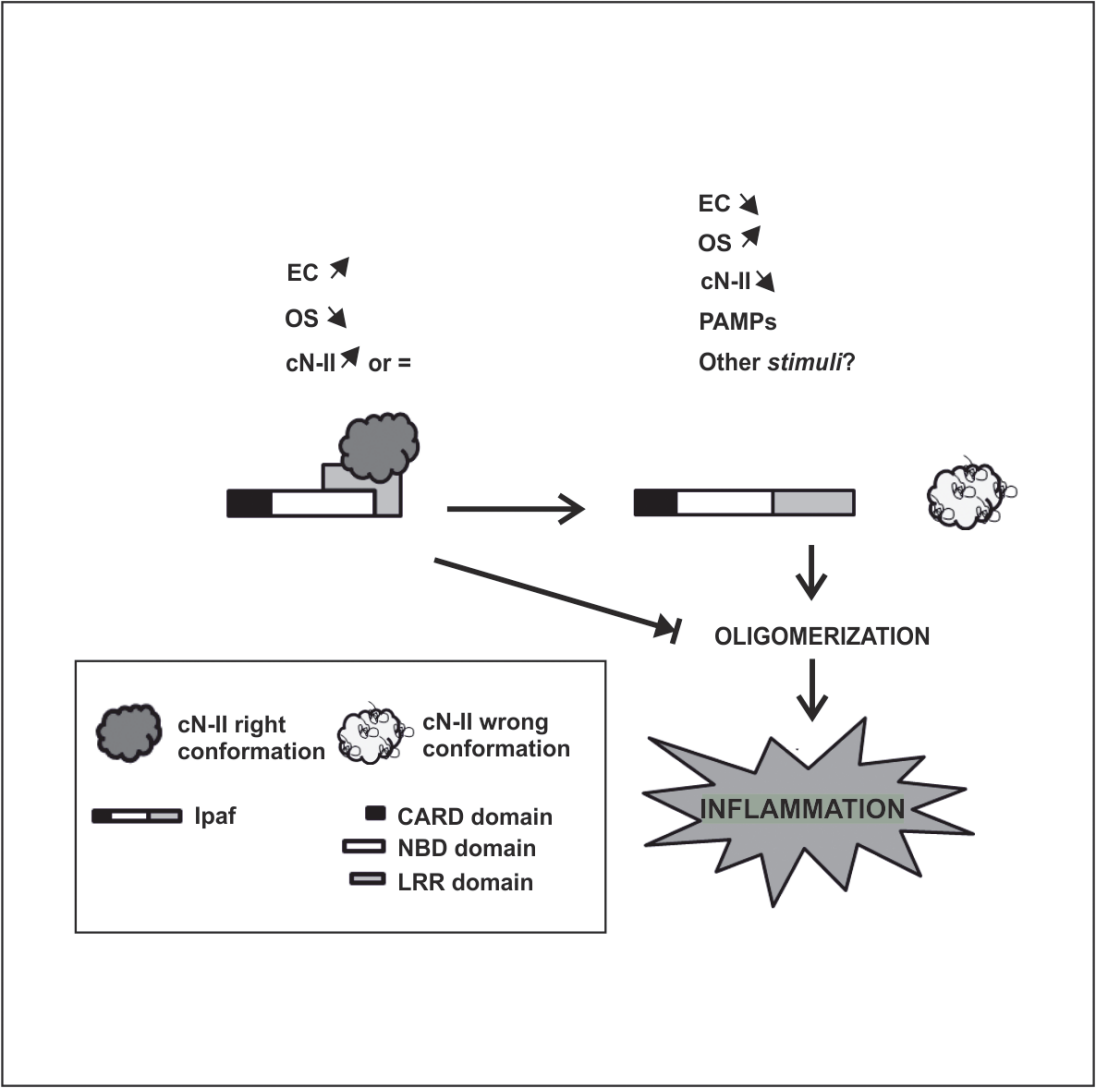
Model representing a possible explanation of the physiological role of cN-II and Ipaf interaction. Left: In presence of a high energy charge (EC), low oxidative stress (OS) and a high or normal expression level of cN-II, Ipaf is maintained in an inactivated configuration due to its interaction with cN-II and the LRR domain. Right: Upon a decrease in EC or in cN-II expression level, an increased OS, the presence of Pathogen Associated Molecular Pattern molecules (PAMPs) or other unidentified stimuli, the cN-II is detached from Ipaf and the LRR domain is free and Ipaf can oligomerize.

Concerning the functionality of the demonstrated interaction, we did not obtain any results but can propose a hypothesis. Ipaf, upon detection by the LRR domain of a wide variety of stress molecules, undergoes oligomerization through the NBD domain followed by recruitment and activation of caspases through the CARD domain [46–49]. In addition, the LRR domain of Ipaf negatively regulates its apoptosis-inducing function through intramolecular interaction with a region comprised between the CARD and NBD domains [23]. This auto-inhibitory mechanism of Ipaf has been recently structurally investigated and confirmed [50]. Thus, we hypothesized that, in the absence of stress molecules or other *stimuli*, Ipaf is maintained in an inactive form (Fig. 7). The evidence that interaction with cN-II was detected in cell lines in the absence of *stimuli* usually leading to Ipaf activation supports the idea that this interaction occurs in the cytoplasm of tumor cell when Ipaf is inactive. Therefore, we can hypothesize that cN-II can contribute to keep the inflammatory response below alarming levels by preventing Ipaf oligomerization through interaction between cN-II and the LRR domain of Ipaf (Fig. 7). Interestingly, cN-II activity and structure depends on the energy charge of the cell [6] as well as the oxidative stress [28]. We can therefore hypothesize a role of cN-II as a sensor of cellular health since the maintenance of its active conformation requires a high energy charge and a reducing environment. As a consequence, when nucleoside triphosphate levels and thus the energy charge decrease and oxidative stress increases, the structural conformation of cN-II is modified, which could release Ipaf, leaving it free to oligomerize and activate the innate inflammatory response. In this case we propose to consider Ipaf as a pattern recognition receptor which could arise to sterile inflammation. Furthermore, a high expression of cN-II mRNA and protein has been associated to a high-proliferative cellular phenotype which, among many probable consequences, makes cells more invasive and thus resistant to many antimetabolites used to inhibit proliferation [30, 33–37]. Thus, we may explain this phenomenon by a saturation of Ipaf due to high level of cN-II expression which may inhibit the oligomerization of the inflammasome by avoiding the release of the LRR from the NBD domain. Furthermore, a partial silencing of cN-II in ADF cultured cells was shown to lead to activation of caspase 3 and consequent apoptosis [17]. This observation is in line with our hypothesis on an inhibitory role of Ipaf by cN-II and explains why the enzyme is always expressed at a low but constant level in many cell types [7].

The originality of our hypothesis is that activation of the innate immune system could involve enzymatic proteins such as cN-II which are likely to be sensors of cellular wellness. Finally, the interaction between cN-II and Ipaf has been consistently demonstrated in this paper and opens for the consideration of new horizons in cell biology based on yet poorly understood roles of these proteins.

## Supporting Information

S1 Fig Expression of recombinant Ipaf B. The immunoblot was performed on cell extract from *E*. *coli* strain “Rosetta” transformed with pReceiver.B01/Ipaf B.
(TIF)

S2 Fig Immunoblot analysis of cN-II and Ipaf protein expression in different human cell lines. (TIF)

S1 Table Nucleotide sequence and aminoacid sequence of positive clones from the two-hybrid screen. Clones 1–18 were obtained in the first experiments, clones 19–47 in the second.
(PDF)
