# Supplementary material for: Cytosolic 5’-Nucleotidase II Interacts with the Leucin Rich Repeat of NLR Family Member Ipaf
Source: PLoS One. 2015 Mar 26;10(3):e0121525. doi: 10.1371/journal.pone.0121525 (PMC4374842; doi:10.1371/journal.pone.0121525)
Supplement: S1 Table — Clones 1–18 were obtained in the first experiments, clones 19–47 in the second. (PDF) [file pone.0121525.s003.pdf]

Clone	Nucleotide sequence	Aminoacid sequence	Protein ID
Exp 1			
1	GGGGACTGA	GD*	
2	GGGTATTATTATTATTACAGTTGTTATTGTTGTTTTTGTTGTTATTATTATTTGGGGTTTCTTGTGTTCTTTCTTTGCGACTCTCCACACTAA	GYYYYYSCYCCFCCYYYLGFLVFFLCDSPH*	
3	GGGGGATACAAAATAATGTACGAAAATTAG	GGYKIMYEN*	
4	GGGAGTGAACATTACATTTTCAGAATAGATCCTAATATTTTATTGAGGGCCTATGTGCTAAAAACTATGCATATCTATATATTGGCCAATTATCTTTAA	GSEHYIFRIDPNILLRAYVLKTMHIYILANYL*	
5	GGGAAATGA	GK*	
6	GGGTGGAGAGTTTAA	GWRV*	
7	GGGGTAAAGTGGTGA	GVKW*	
8	GGGTCGACAACTGCCAGCTAA	GSTTAS*	
9	GGGAGGAAAATATTATTAAATTTATATCCCTAA	GRKILLNLYP*	
10	GGGGAGACAATGGGGTTTTCTAAATATACAATACATGTCGTCCGCACACAGGGACAATTTGACTTAATTTGGAAGAAATTTTGA	GETMGFSKYTIHVVRTQGQFDLIWKKF*	
11	GGGAATAAAGTGCAGGAGAGAGAGTTGTTGGCCCTGGGGAGCGAGTAG	GNKVQERELLALGSE*	
12	GGGAGGTAA	GR*	
13	GGGTAG	G*	
14	GGGTGCAGAAGGAAATATTTGAGCAGCCAGAGTCTGTCGTGA	GCRRKYLSSQSLS*	
15	GGGATTGAAGTGGTGGAGCTGCTGCTAGATAAAGGTGCTAAAGTGTCTGCTGTAGATAAGAAAGGAGATACTCCCTTGCATATTGCTATTCGTGGAAGGAGCCGGAAACTGGCAGAACTGCTTTTAAGAAATCCCAAAGATGGGCGATTACTTTATAGGCCCAACAAAGCAGGCGAGACTCCTTATAATATTGACTGTAGCCATCAGAAGAGTATTTTAACTCAAATATTTGGAGCCAGACACTTGTCTCCTACTGAAACAGACGGTGACATGCTTGGATATGATTTATATAGCAGTGCCCTGGCAGATATTCTCAGTGAGCCTACCATGCAGCCACCCATTTGTGTGGGGTTATATGCACAGTGGGGAAGTGGGAAATCTTTCTTACTCAAGAAACTAGAAGACGAAATGAAAACCTTCGCCGGACAACAGATTGAGCCTCTCTTTCAGTTCTCATGGCTCATAGTGTTTCTTACCCTGCTACTTTGTGGAGGGCTTGGTTTATTGTTTGCCTTC	GIEVVELLLDKGAKVSAVDKKGDTPLHIAIRGRSRKLAELLLRNPKDGRLLYRPNKAGETPYNIDCSHQKSILTQIFGARHLSPTETDGDMLGYDLYSSALADILSEPTMQPPICVGLYAQWGSGKSFLLKKLEDEMKTFAGQQIEPLFQFSWLIVFLTLLLCGGLGLLFAF*	KIDINS220
16	GGGGTAACTTTAAAAAAACATCCCTCAGGTCCCGATATATTTCCTTGGATTCATTTCACTTGGCTAGAAATTACACTGTGCTCAATGCCTTAA	GVTLKKHPSGPDIFPWIHFTWLEITLCSMP*	
17	GGGGCCCTGGGTTCAGCTGTTAGTTTCGCTCTTTTGTATCTTGCTGATGCTGTTAGGTTTCTCTGTTTCAAAGGTCAGCATTCTGCTAATTAA	GALGSAVSFALLYLADAVRFLCFKGQHSAN*	
18	GGGGGCAGGAGCGGCGACCTTTGA	GGRSGDL*	
Exp 2			
19	GGGGACTGA	GD*	
20	GGGGGATACAAAATAATGTACGAAAATTAG	GGYKIMYEN*	
21	GGGAGTGAACATTACATTTTCAGAATAGATCCTAATATTTTATTGAGGGCCTATGTGCTAAAAACTATGCATATCTATATATTGGCCAATTATCTTTAATAA	GSEHYIFRIDPNILLRAYVLKTMHIYILANYL*	

22	GGGGTTCTATTTTTCAATCATCAAAAAGTAATTATAAATACGTATTACAAACAAGTGGATGTTTTTAATGACCAATTGAGTAAGAACATCCCTGTCTTAACTGGCCTAAATTTCTTCTGGTAG	GVLFFNHQKVIINTYYKQVDVFNDQLSKNIPVLTGLNFFW*	
23	GGGAAATGA	GK*	
24	GGGAACGCTTAA	GNA*	
25	GGGTGGAGAGTTTAA	GWRV*	
26	GGGGTAAAGTGGTGA	GVKW*	
27	GGGATCTTGAAAAAATGTAATTTATTTTTGATAACGGCTCTTAAACTTTAA	GILKKCNLFLITALKL*	
28	GGGGATTCTAGTACAAAGTTACTGTTTAACAAAAGCAACATAAACTCTGGGAAAGATTTCATTTTGCCATGTTATATTTACTGTTTATTCTGTGTACTAGTACATATCTTTAAATTACCAAAAAACAAGAAACAAAACATAAAAACCCCAAAACTATCACTTGGAATTAGCAATATCACCCAACTGGCTTTAAAATTGAAAATTTAA	GDSSTKLLFNKSNINSGKDFILPCYIYCLFCVLVHIFKLPKNKKQNIKTPKLSLGISNITQLALK*	
29	GGGTCGACAACTGCCAGCTAA	GSTTAS*	
30	GGGAGGAAAATATTATTAAATTTATATCCCTAA	GRKILLNLYP*	
31	GGGGAGACAATGGGGTTTTCTAG	GGDNGVF*	
32	GGGGCAGCAAACGTTGTTTAA	GAANVV*	
33	GGGAATAAAGTGCAGGAGAGAGAGTTGTTGGCCCTGGGGAGCGAGTAG	GNKVQERELLALGSE*	
34	GGGGGCGGCCACCAGTGGAACTGA	GGGHQWN*	
35	GGGAGGTAA	GR*	
36	GGGTAG	G*	
37	GGGGGAAGCCTCTGTCAGCTCAGCCTCCAAAGGAGCCAGCCTCTCCCCAGTTCCTGA	GGSLCQLSLQRSQPLPSS*	
38	GGGTGCAGAAGGAAATATTTGAGCAGCCAGAGTCTGTCGTGA	GCRRKYLSSQSLS*	
39	GGGATTGAAGTGGTGGAGCTGCTGCTAGATAAAGGTGCTAAAGTGTCTGCTGTAGATAAGAAAGGAGATACTCCCTTGCATATTGCTATTCGTGGAAGGAGCCGGAAACTGGCAGAACTGCTTTTAAGAAATCCCAAAGATGGGCGATTACTTTATAGGCCCAACAAAGCAGGCGAGACTCCTTATAATATTGACTGTAGCCATCAGAAGAGTATTTTAACTCAAATATTTGGAGCCAGACACTTGTCTCCTACTGAAACAGACGGTGACATGCTTGGATATGATTTATATAGCAGTGCCCTGGCAGATATTCTCAGTGAGCCTACCATGCAGCCACCCATTTGTGTGGGGTTATATGCACAGTGGGGAAGTGGGAAATCTTTCTTACTCAAGAAACTAGAAGACGAAATGAAAACCTTCGCCGGACAACAGATTGAGCCTCTCTTTCAGTTCTCATGGCTCATAGTGTTTCTTACCCTGCTACTTTGTGGAGGGCTTGGTTTATTGTTTGCCTTCACGGTCCACCCAAATCTTGAAATAGCAGTGTCACTGAGCTTCTTGGCTCTCTTATATATATTCTTTATTGTCATTTACTTTGGTGGACGAAGAGAAGGAGAGAGTTGGAATTGGGCCTGGGTCCTCAGCACTAGATTGGCAAGACATATTGGATATTTAGAACTCCTCCTTAAATTGATGTTTGTGAATCCACCTGAGTTGCAGAGCAGACTACTAAAGCTTTACCTGGGAGGTTTTTGTTTACAGATACATAGACTGTCCAGTGTAGTGGAG	GIEVVELLLDKGAKVSAVDKKGDTPLHIAIRGRSRKLAELLLRNPKDGRLLYRPNKAGETPYNIDCSHQKSILTQIFGARHLSPTETDGDMLGYDLYSSALADILSEPTMQPPICVGLYAQWGSGKSFLLKKLEDEMKTFAGQQIEPLFQFSWLIVFLTLLLCGGLGLLFAFTVHPNLEIAVSLSFLALLYIFFIVIYFGGRREGESWNWAWVLSTRLARHIGYLELLLKLMFVNPPELQSRLLKLYLGGFCLQIHRLSSVVE*	KIDINS220
40	GGGGCATTTTTTGGAAAGAACCCTCTGAAAAACTCC	GAFFGKNPLKNSQQLNLAGNRVSSDG	Ipaf

	CAGCAGTTGAATTTGGCGGGAAATCGTGTGAGCAGTGATGGATGGCTTGCCTTCATGGGTGTATTTGAGAATCTTAAGCAATTAGTGTTTTTTGACTTTAGTACTAAAGAATTTCTACCTGATCCAGCATTAGTCAGAAAACTTAGCCAAGTGTTATCCAAGTTAACTTTTCTGCAAGAAGCTAGGCTTGTTGGGTGGCAATTTGATGATGATGATCTCAGTGTTATTACAGGTGCTTTTAAACTAGTAACTGCATAA	WLAFMGVFENLKQLVFFDFSTKEFLPDPALVRKLSQVLSKLTFLQEARLVGWQFDDDDLSVITGAFKLVTA*	
41	GGGGTAACTTTAAAAAAACATCCCTCAGGTCCCGATATATTTCCTTGGATTCATTTCACTTGGCTAGAAATTACACTGTGCTCAATGCCTTAA	GVTLKKHPSGPDIFPWIHFTWLEITLCSMP*	
42	GGGGCCCTGGGTTCAGCTGTTAGTTTCGCTCTTTTGTATCTTGCTGATGCTGTTAGGTTTCTCTGTTTCAAAGGTCAGCATTCTGCTAATTAA	GALGSAVSFALLYLADAVRFLCFKGQHSAN*	
43	GGGGGCAGGAGCGGCGACCTTTGA	GGRSGDL*	
44	GGGTACGTATTTTAG	GYVF*	
45	GGGGATTCTATGAAAAGCCAAGCCATCTTGAGAAGTCATGTGTAG	GDSMKSQAILRSHV*	
46	GGGACCAACGTGAAAAGTGCCATTTTTAGAATAACTTTAAAGCTTAACAGGTTTCCTTTTAATATCCTTTTTTTGTGTGCTCTTTACTTACACAATGGCTTTGTTTTGCTTTTTCAGCCACACCCCTTATGTGAACTAGTGCCTTTGGGTATCACGTAA	GTNVKSAIFRITLKLNRFPFNILFLCALYLHNGFVLLFQPHPLCELVPLGIT*	
47	GGGGACGCCGCGTCACGAGTCAGCCAAAGATGGCTGCGCCCAGGTAATTTGAGCAAAGGCCACAGTGAACTCCGGCGTGGCTGA	GDAASRVSQRWLRPGNLSKGHSELRRG*	
